# Factors Influencing the Behavioral Intentions and Use Behaviors of Telemedicine in Patients With Diabetes: Web-Based Survey Study

**DOI:** 10.2196/46624

**Published:** 2023-12-28

**Authors:** Huige Shao, Chaoyuan Liu, Li Tang, Bian Wang, Hebin Xie, Yiyu Zhang

**Affiliations:** 1 Department of Endocrinology, The Affiliated Changsha Central Hospital Hengyang Medical School University of South China Changsha China; 2 Department of Oncology The Second Xiangya Hospital Central South University Changsha China; 3 Science and Education Department, The Affiliated Changsha Central Hospital Hengyang Medical School University of South China Changsha China

**Keywords:** diabetes mellitus, telemedicine, survey, China, behavioral intention, acceptance, technology, technology use, diabetic, outpatient, eHealth, remote care, older adult patients, low income, diabetes, type 1, type 2

## Abstract

**Background:**

Telemedicine has great potential for diabetes management. The COVID-19 pandemic has boosted the development of telemedicine. However, the factors influencing the behavioral intentions to use and use behaviors of telemedicine in patients with diabetes in China are not clear.

**Objective:**

We aimed to understand the determinants of behavioral intention to use telemedicine based on an extended Unified Theory of Acceptance and Use of Technology model and to identify demographic factors associated with telemedicine use in patients with diabetes in China.

**Methods:**

Patients with diabetes who are aged ≥18 years were surveyed from February 1 to February 7, 2023. We distributed the survey link in 3 WeChat groups including a total of 988 patients with diabetes from the outpatient department or patients discharged from Changsha Central Hospital. Structural equation modeling was used to understand the determinants of behavioral intention. A multivariate logistic regression analysis was used to identify the demographic factors associated with telemedicine use.

**Results:**

In total, 514 questionnaires were collected. Of the respondents, 186 (36.2%) were diagnosed with COVID-19. The measurement model showed acceptable reliability, convergent validity, discriminant validity, and data fit indices. The model explained 63.8% of the variance in behavioral intention. Social influence, performance expectancy, and facilitating conditions positively influenced behavioral intention (β=.463, *P*<.001; β=.153, *P*=.02; and β=.257, *P*=.004, respectively). Perceived susceptibility, perceived severity, and effort expectancy had no significant impact on behavioral intention (all *P*>.05). The overall use of telemedicine was 20.6% (104/514). After adjusting for the behavioral intention score, the multivariate regression analysis showed that age, education, and family income were associated with telemedicine use. Telemedicine use was higher in the 40 to 59 years and 18 to 39 years age groups than in the ≥60 years age group (odds ratio [OR] 4.35, 95% CI 1.84-10.29, *P*=.001; OR 9.20, 95% CI 3.40-24.88, *P*<.001, respectively). Telemedicine use was higher in the senior high school and the university and more groups than in junior high school education and less group (OR 2.45, 95% CI 1.05-5.73, *P*=.04; OR 2.63, 95% CI 1.11-6.23, *P*=.03, respectively). Patients with a higher family income used telemedicine more often than the patients who had an annual family income ≤¥10,000 (CNY ¥1=US $0.1398; ¥10,000-¥50,000 group: OR 3.90, 95% CI 1.21-12.51, *P*=.02; ¥50,000-¥100,000 group: OR 3.91, 95% CI 1.19-12.79, *P*=.02; >¥100,000 group: OR 4.63, 95% CI 1.41-15.27, *P*=.01).

**Conclusions:**

Social influence, performance expectancy, and facilitating conditions positively affected the behavioral intention of patients with diabetes to use telemedicine. Young patients, highly educated patients, and patients with high family income use telemedicine more often. Promoting behavioral intention and paying special attention to the needs of older adult patients, patients with low income, and patients with low levels of education are needed to encourage telemedicine use.

## Introduction

### Background

The prevalence of diabetes has been increasing worldwide [1]. In 2018, the estimated prevalence of diabetes among adults in China was 10.9%, representing more than 100 million adults [2]. However, only 32.9% of patients were treated, and only 50.1% of patients receiving treatment had adequate glycemic control [2]. Poor glycemic control can cause various complications and impose a heavy economic burden on the country. Telemedicine, which provides remote consultation, diagnosis, and prescriptions over computers and smartphones, ensures quick physician-patient interaction across the barriers of distance and time. The most common modalities of telemedicine include real-time technology, store-and-forward technology, remote monitoring, and mobile health (mHealth) approaches [3]. With the development of mobile apps and wearable devices, telemedicine shows great potential for diabetes management. Studies have shown that telemedicine, such as mobile apps for diabetes management, is effective for glycemic control in patients with diabetes, especially patients in remote areas [4-6]. The COVID-19 pandemic has boosted the development of telemedicine. During the COVID-19 pandemic, telemedicine was used to reduce patients’ office consultations, prevent overcrowding in hospitals, facilitate patient and physician communication and cooperation, and save travel time. To mitigate the impact of COVID-19 on population health, many countries, including China, have promoted telemedicine as a solution for health care professionals to continue offering medical services to their patients [7]. China released the first Expert Consensus on Telemedicine Management of Diabetes in 2020 [8]. Studies have also suggested that telemedicine can effectively reduce the impact of COVID-19 isolation on glycemic control in patients with diabetes [9-11].

Compared with other COVID-19 variants of concern, the Omicron variant is characterized by significantly greater infectivity and lower severity of human infections [12]. Thus, on December 7, 2022, China lifted most of its zero tolerance COVID-19 restrictions [13]. Since then, people have been able to visit hospitals without COVID-19 restrictions or choose telemedicine. At this time, people’s behavioral intentions (BIs) to use and use behaviors of telemedicine were more closely tied to their post–COVID-19 situations. However, in this specific context, patients’ telemedicine use behaviors are unclear. People’s use behaviors for a certain technology often depend on their intentions to use it. Several studies have applied umbrella theoretical models to understand the determinants of use intentions for mHealth services [14,15]. One of the most frequently used theoretical models is Unified Theory of Acceptance and Use of Technology (UTAUT), which was developed by Venkatesh et al [16]. The UTAUT model integrates the 8 existing models, including the technology acceptance model, theory of rational action, theory of planned behavior, technology acceptance model and theory of planned behavior combined, motivation model, PC use model, diffusion of innovation theory, and social cognitive theory, and it outperforms them in terms of explanatory power. Since its introduction, the UTAUT model has been applied in multiple domains [17-19]. However, a theoretical model must be identified and tested for various technologies and in different user groups to provide a context-related understanding of technology adoption [16]. During the outbreak of the COVID-19 epidemic, people’s BIs and use behaviors of telemedicine, as well as their influencing factors, may change.

Furthermore, although intention to use is a determinant of use behavior, there is usually a gap between BI to use and actual use [20]. Studies have found that demographic characteristics such as sex, age, family income, and education level are associated with telemedicine use [21-23]. However, it is not clear whether the difference in the use of telemedicine is due to the difference in the BIs of patients with different demographic characteristics. After adjusting for BIs to use telemedicine, it is unclear whether these associations remain. Understanding the differences in telemedicine use among patients with different demographic characteristics will help to develop measures to promote the development of telemedicine in the post–COVID-19 pandemic era.

### Objectives

To understand the differences in telemedicine use among patients with diabetes to promote the use of telemedicine in the post–COVID-19 pandemic era, we first analyzed the determinants of patients’ BIs to use telemedicine through a theoretical model and then adjusted the BIs through multiple regression analysis to analyze the associations between telemedicine use and demographic characteristics.

### Research Model and Hypotheses

According to the UTAUT model, performance expectancy (PE), effort expectancy (EE), and social influence (SI) are the core determinants of BI to use and facilitating conditions (FCs) and BIs to use are direct determinants of use behavior. Venkatesh et al [24] proposed the updated UTAUT2 in a consumer information technology context and found a direct association between FCs and BIs.

PE is defined as the degree to which individuals perceive that a new technology will help them attain gains in task performance [16]. In this study, PE indicates people’s perceptions of the usefulness of telemedicine for health management. Several studies have shown that PE is a major determinant of the BI to use mHealth services [25-27]. Unless patients with diabetes think telemedicine is useful for them, they will not use it. Thus, we proposed the following hypothesis:

- *Hypothesis 1: PE positively influences the BIs of patients with diabetes to use telemedicine.*

EE is defined as “the subjective perception of the difficulty of a system” [16]. If patients perceive certain technologies to be easy to use, they tend to use them. This hypothesis has been tested in many studies, especially among older adults [15]. Therefore, we proposed the following hypothesis:

- *Hypothesis 2: EE positively influences the BIs of patients with diabetes to use telemedicine.*

SI is defined as the extent to which people think that others who are important to them or who can influence their behaviors think that they should use a specific technology [24]. Regarding health care, patients’ intentions to adopt a health behavior are often influenced by the opinions of their health care professionals, other patients with the same disease, and their family members. Therefore, we proposed the following hypothesis:

- *Hypothesis 3: SI positively influences diabetes patients’ intentions to use telemedicine.*

FCs are defined as “the degree to which an individual believes that an organization and technical infrastructure exist to support the use of a system” [16]. In this study, FCs indicate the subjective perception of the support and resources available to support the use of telemedicine. Although the original UTAUT did not find a direct association between FCs and BI, UTAUT2 and several other studies concerning information technologies demonstrated this relationship [18,24,28,29]. Facilitation available to each patient can vary significantly across telemedicine devices, network access, and human resource support. Thus, we proposed the following hypothesis:

- *Hypothesis 4: FCs positively influence the BI of patients with diabetes to use telemedicine.*

Context is the environment in which a technology is used, and it may affect an individual’s BIs [30]. According to the health belief model, individuals will not take health-related actions unless they feel susceptible to or experience the severity of a disease [31]. During the COVID-19 pandemic, telemedicine reduced face-to-face contact to control the risk of COVID-19 infection. Therefore, we proposed the following hypothesis:

- *Hypothesis 5: Perceived susceptibility to COVID-19 positively influences the BI of patients with diabetes to use telemedicine.*
- *Hypothesis 6: Perceived severity (PSE) of COVID-19 positively influences the BI of patients with diabetes to use telemedicine.*

### Demographic Factors

Previous studies have shown that demographic characteristics such as sex, age, education, and family income were associated with telemedicine use [21-23]. We argue that these demographic characteristics may affect the BI toward telemedicine. Thus, we adjusted for sex, age, education, and family income in the model.

**Figure 1:**
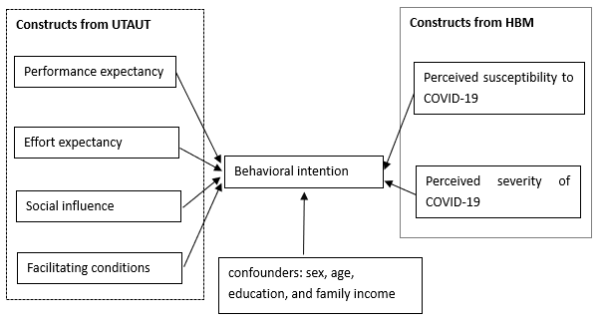
Research model. HBM: Health Belief Model; UTAUT: Unified Theory of Acceptance and Use of Technology.

The research hypotheses are summarized in the research model (Figure 1).

## Methods

### Survey Instrument

All survey items (Textbox 1) were adopted from scales validated in previous studies and modified to adapt them to telemedicine in the context of diabetes and COVID-19. The questionnaire was translated by 2 native Chinese speakers proficient in English. A pilot study was conducted in a sample of 20 patients with diabetes from the outpatient department of Changsha Central Hospital, and the participants were asked to provide feedback on the conciseness and clarity of the questions. A 5-point Likert scale was used for all items, with “1” representing “strongly disagree” and “5” representing “strongly agree.” Demographic information such as age, sex, annual family income, residence, type of diabetes, diabetes history, and education level were also collected.

**Measurement items of the constructs.**

**Performance expectancy (PE) [15,32,33]**

- PE1: Telemedicine can reduce my risk of getting a COVID-19 infection (new).
- PE2: Telemedicine can save my time.
- PE3: Telemedicine can save money (new).
- PE4: Telemedicine enables me to be effectively treated.
- PE5: Overall, telemedicine is useful to me.

**Effort expectancy (EE) [15,32,33]**

- EE1: My interaction with telemedicine is clear and understandable.
- EE2: Learning how to use telemedicine is easy for me.
- EE3: I find telemedicine easy to use.

**Social influence (SI) [15,32,33]**

- SI1: People whose opinions I value (eg, my doctors) think I should use telemedicine.
- SI2: People who influence my behavior (eg, peers with diabetes) think I should use telemedicine.
- SI3: People who are important to me (eg, family members) think I should use telemedicine.

**Facilitating condition (FC) [24,32,34]**

- FC1: I have the resources (eg, network) necessary to use telemedicine.
- FC2: I have the knowledge necessary to use telemedicine (eg, how to find a telemedicine platform).
- FC3: I can get help from others when I have difficulties using telemedicine.

**Perceived susceptibility (PSU) [35]**

- PSU1: I'm worried about the likelihood of getting COVID-19.
- PSU2: I think we patients with diabetes are more likely to be infected with COVID-19.
- PSU3: Overall, getting COVID-19 is possible for me.

**Perceived severity (PSE) [14]**

- PSE1: I'm worried I will be very sick if I get COVID-19.
- PSE2: I think we patients with diabetes will be more seriously ill if we get COVID-19.
- PSE3: I'm worried it will be very serious if I get COVID-19.

**Behavioral intention (BI) [15,32,33]**

- BI1: I intend to use or continue to use telemedicine.
- BI2: I plan to use telemedicine frequently.
- BI3: Overall, I have a high intention to use telemedicine.

### Samples and Survey Methods

The participants were patients with diabetes in China who were aged ≥18 years. Convenience sampling was used. The web-based survey tool Sojump (Changsha ran Xing InfoTech Ltd) was used to collect data. From February 1 to February 7, 2023, we distributed the survey link in 3 WeChat groups consisting of 988 outpatients with diabetes from the outpatient department or patients discharged from Changsha Central Hospital, which is a large public tertiary hospital with more than 2000 beds in Changsha City, mainly treating patients from Hunan Province. From November 2021 to February 2023, we recruited patients with diabetes who had been treated in Changsha Central Hospital into our 3 WeChat groups after they provided informed consent to facilitate follow-up. The survey links were distributed in the 3 WeChat groups. Before the survey, we introduced its purpose and explained the definition of telemedicine. After obtaining consent, the survey continued. Each mobile IP address could complete the questionnaire only once. To increase the response rate, we reminded all patients in the groups to complete the survey. Questionnaires completed in ≤2 minutes and those completed by patients aged ≤18 years were excluded. No compensation was provided for participation in the survey.

### Ethics Approval

The study was approved by the ethics committee of South China University’s affiliated Changsha Central Hospital (ID: 2022-S0217).

### Data Analysis

A descriptive analysis was performed to summarize the participants’ sociodemographic characteristics. Continuous variables are expressed as mean (SD) or median (IQR), where appropriate. Categorical variables are expressed as number (percentage). SPSS (version 23.0; IBM Corp) via maximum likelihood estimation was used to analyze the collected data. In addition, SPSS Amos (version 23.0) was used to conduct structural equation modeling and test the proposed research model. Before evaluating the structural model, we assessed the measurement model to evaluate construct reliability, convergent validity, discriminant validity, and data fit indices.

Differences among groups were assessed using the chi-square test or independent 2-tailed *t* tests. Telemedicine use was an observable variable. In our study, telemedicine use behavior was a binary dependent variable, which was not suitable for structural equation modeling. Thus, in the second part, we used logistic regression to investigate the relationships among sex, age, education level, family income, residence, disease information, BI, and the use of telemedicine. The sample size estimation was based on the use of telemedicine in the study and on the principle of 10 outcome events per variable [36]. As there is no literature on the use of telemedicine in China, using an estimated use of telemedicine of 20% in the pilot survey and 10 variables, we aimed to enroll at least 500 samples. On the basis of expertise, we set a BI score of ≤10 as low BI and ≥10 as high BI. The sample was divided into 2 groups according to the total BI score (low BI group <10; high BI group ≥10). We performed a univariable analysis to obtain unadjusted odds ratios (ORs) of potential correlates of telemedicine use with demographic factors, disease characteristics, and BI. We then entered all the variables in the multivariate analysis to obtain the multivariable adjusted ORs. Statistical significance was set at *P*<.05.

## Results

### Sample Characteristics

In total, 42 questionnaires completed in ≤2 minutes or completed by patients aged ≤18 years were eliminated and 514 qualified questionnaires were collected. Of the respondents, 273 (53.1%) were male and 241 (46.9%) were female. A total of 465 (90.5%) respondents had been vaccinated for COVID-19 and 186 (36.2%) respondents had been infected with COVID-19. The demographic characteristics of qualified participants are shown in Table 1.

**Table 1:** Characteristics of the total sample (N=514).

Characteristics		Sample, n (%)	Use of telemedicine, n (%)	*P* value^a^	
**Sex**					.01
	Male	273 (53.1)	44 (16.1)		
	Female	241 (46.9)	60 (24.9)		
**Age (y)**					<.001
	≥60	138 (26.8)	7 (5.1)		
	40-59	268 (52.1)	56 (20.9)		
	18-39	108 (21.0)	41 (38.0)		
**Education**					<.001
	Junior middle school or less	118 (23)	10 (8.5)		
	High school	156 (30.4)	29 (18.6)		
	University or more	240 (46.7)	65 (27.1)		
**Diabetes history (y)**					.46
	<1	81 (15.8)	21 (25.9)		
	1-5	156 (30.4)	33 (21.2)		
	6-10	123 (23.9)	23 (18.7)		
	>10	154 (30.0)	27 (17.5)		
**Residence**					.86
	Urban	412 (80.2)	84 (20.4)		
	Rural	102 (19.8)	20 (19.6)		
**Annual family income (¥^b^)**					.002
	<10,000	66 (12.8)	4 (6.1)		
	10,000-50,000	165 (32.1)	30 (18.2)		
	50,000-100,000	146 (28.4)	31 (21.2)		
	>100,000	137 (26.7)	39 (28.5)		
**Diabetes type**					.006
	Type 1 diabetes mellitus	83 (16.1)	24 (28.9)		
	Type 2 diabetes mellitus	373 (72.6)	62 (16.6)		
	Others	17 (3.3)	7 (14.2)		
	Unknown	41 (8.0)	11 (26.8)		
**Vaccinated for COVID-19**					.28
	No	49 (9.5)	7 (14.3)		
	Yes	465 (90.5)	97 (20.9)		
**COVID-19 infection**					.22
	No	328 (63.8)	61 (18.6)		
	Yes	186 (36.2)	43 (23.1)		

^a^*P* values were calculated using the chi-square test.

^b^CNY ¥1=US $0.1398.

Of the 514 patients, the overall use of telemedicine was 104 (20.6%). Usage in female patients was higher than that in male patients (60/241, 24.9% vs 44/273, 16.1%; *P*=.01). Telemedicine use was higher among younger patients than among patients aged ≥60 years (18-39 y vs 40-59 y vs ≥60 y: 41/108, 38% vs 56/268, 20.9% vs 7/138, 5.1%; *P*<.001). Patients with higher education levels had a higher usage of telemedicine (junior middle school or less vs high school vs university or more: 10/118, 8.5% vs 29/156, 18.6% vs 65/240, 27.1%, *P*<.001, respectively). Patients with higher family incomes used telemedicine more often than those with low family incomes (*P*=.002). There was no significant correlation between telemedicine use and whether the patients had been vaccinated for COVID-19 or infected with COVID-19.

The main concerns of the patients regarding telemedicine included effectiveness (231/514, 44.9%), security (127/514, 24.7%), privacy (61/514, 11.9%), cost (26/514, 5.1%), and other reasons (69/514, 13.4%).

### Measurement Model

The factor loadings of each item were above the recommended value of 0.6 [37]. Reliability was assessed using Cronbach α. Composite reliability of 0.7 is an indicator of acceptable internal consistency. Convergent validity was assessed using the average variance extracted (AVE). Constructs with Cronbach α>.7 and AVE >0.5 were considered acceptable [38]. As shown in Table 2, all the constructs demonstrated acceptable levels of reliability and validity.

Discriminant validity is the degree to which each construct measures different variables. Discriminant validity is established if the AVE values of each construct are greater than the squared correlation coefficient between the constructs [39,40]. Consequently, the data in Table 3 demonstrate an acceptable level of discriminant validity.

The model fit was generally considered acceptable when the root mean square error of approximation values were below 0.05; the ratio of chi-square and df was below 3; and the adjusted goodness-of-fit index, goodness-of-fit index, comparative fit index, and normed fit index were above 0.90 [14]. Table 4 indicates that the fit indices of the research model were acceptable.

**Table 2:** Results of the measurement model.

Constructs and items			Factor loadings		Score, mean (SD)		AVE^a^			CR^b^			Cronbach α	
**PSE^c^**								0.698			0.873			.855
	PSE1	0.899		3.47 (0.86)										
	PSE2	0.867		3.62 (0.91)										
	PSE3	0.731		3.26 (0.91)										
**PSU^d^**								0.691			0.870			.747
	PSU1	0.801		3.63 (0.93)										
	PSU2	0.881		3.41 (0.90)										
	PSU3	0.809		3.74 (0.85)										
**PE^e^**								0.528			0.846			.845
	PE1	0.608		3.65 (0.88)										
	PE2	0.717		3.85 (0.72)										
	PE3	0.651		3.38 (0.78)										
	PE4	0.750		3.45 (0.75)										
	PE5	0.878		3.73 (0.72)										
**EE^f^**								0.775			0.911			.892
	EE1	0.892		3.41 (0.82)										
	EE2	0.808		3.41 (0.85)										
	EE3	0.936		3.40 (0.77)										
**SI^g^**								0.758			0.904			.903
	SI1	0.872		3.62 (0.78)										
	SI2	0.872		3.56 (0.77)										
	SI3	0.867		3.79 (0.71)										
**FC^h^**								0.545			0.781			.781
	FC1	0.699		3.63 (0.71)										
	FC2	0.679		3.85 (0.65)										
	FC3	0.828		3.56 (0.72)										
**BI^i^**								0.797			0.921			.922
	BI1	0.945		3.55 (0.76)										
	BI2	0.817		3.44 (0.75)										
	BI3	0.911		3.39 (0.75)										

^a^AVE: average variance extracted.

^b^CR: composite reliability.

^c^PSE: perceived severity.

^d^PSU: perceived susceptibility.

^e^PE: performance expectancy.

^f^EE: effort expectancy.

^g^SI: social influence.

^h^FC: facilitating condition.

^i^BI: behavioral intention.

**Table 3:** The square root of average variance in the latent variables and correlation coefficient matrix.

Construct	PSE^a^	PSU^b^	PE^c^	EE^d^	SI^e^	FC^f^	BI^g^
PSE	*0.835* ^h^	—^i^	—	—	—	—	—
PSU	0.632	*0.831*	—	—	—	—	—
PE	0.219	0.185	*0.727*	—	—	—	—
EE	−0.004	0.048	0.537	*0.880*	—	—	—
SI	0.143	0.133	0.695	0.613	*0.871*	—	—
FC	0.130	0.105	0.608	0.614	0.696	*0.738*	—
BI	0.072	0.073	0.589	0.492	0.703	0.633	*0.893*

^a^PSE: perceived severity.

^b^PSU: perceived susceptibility.

^c^PE: performance expectancy.

^d^EE: effort expectancy.

^e^SI: social influence.

^f^FC: facilitating condition.

^g^BI: behavioral intention.

^h^Italicized values represent the square root of the average variance extracted; the values below them indicate the correlation coefficients.

^i^Not applicable.

**Table 4:** Fit indexes of the research model.

Fit index	χ^2^:*df*	GFI^a^	AGFI^b^	NFI^c^	CFI^d^	RMSEA^e^	IFI^f^
Research model	2.922	0.912	0.894	0.92	0.930	0.051	0.931
Recommended value	<3	>0.9	>0.9	>0.9	>0.9	<0.05	>0.9

^a^GFI: goodness-of-fit index.

^b^AGFI: adjusted goodness-of-fit index.

^c^NFI: normed fit index.

^d^CFI: comparative fit index.

^e^RMSEA: root mean square error of approximation.

^f^IFI: incremental fit index.

### Structural Model

Overall, the model explained 63.8% of the variance in BI (Table 5). Table 5 shows that SI, PE, and FCs positively influenced BI (β=.463, *P*<.001; β=.153, *P*=.02; and β=.257, *P*=.004, respectively). Perceived susceptibility, PSE, and EE had no significant impact on BI (all *P*>.05). Demographics, including sex, age, education, and family income had no significant impact on BI (all *P*>.05).

**Table 5:** Structural model explaining behavioral intention.

			BI^a^ （*R*^2^=63.8%）		
			β		*P* value
**Hypothesis**					
	PE^b^→BI	.153		.02	
	EE^c^→BI	−.02		.67	
	SI^d^→BI	.463		<.001	
	FC^e^→BI	.257		.004	
	PSU^f^→BI	.014		.73	
	PSE^g^→BI	−.078		.08	
**Confounders**					
	Sex	.015		.62	
	Age (y)	.26		.39	
	Education	−.26		.39	
	Annual family income	−.002		.95	

^a^BI: behavioral intention.

^b^PE: performance expectancy.

^c^EE: effort expectancy.

^d^SI: social influence.

^e^FC: facilitating condition.

^f^PSU: perceived susceptibility.

^g^PSE: perceived severity.

### Factors Associated With the Use of Telemedicine in Patients With Diabetes

The total BI scores of patients who had been vaccinated for COVID-19 showed no difference from those of patients who had not been vaccinated (mean 10.40, SD 2.09 vs mean 10.14, SD 2.28, *P*=.41). Similarly, the total BI scores of the patients who had been infected with COVID-19 showed no difference from those of the patients who had not been infected (mean 10.54, SD 2.01 vs mean 10.29, SD 2.15, *P*=.19).

Univariate logistic regression analysis showed that gender, age, education, family income, diabetes type, and BI score were related to patients’ telemedicine use (Table 6). Then, we entered all the variables in the multivariate analysis to obtain the multivariable adjusted ORs and found that age, education, family income, and BI score were still related to patients’ telemedicine use. The rate of telemedicine use was higher in patients aged 40 to 59 years and those aged 18 to 39 years than in patients aged ≥60 years (OR 4.35, 95% CI 1.84-10.29, *P*=.001; OR 9.20, 95% CI 3.40-24.88, *P*<.001, respectively). The use of telemedicine was higher among the high school group and the university and more group than among the junior middle school education and less group (OR 2.45, 95% CI 1.05-5.73, *P*=.04; OR 2.63, 95% CI 1.11-6.23, *P*=.03, respectively). The patients with a higher family income had a higher use of telemedicine than those with an annual family income of less than ¥10,000 (CNY ¥1=US $0.1398; ¥10,000-¥50,000 group: OR 3.90, 95% CI 1.21-12.51, *P*=.02; ¥50,000-¥100,000 group: OR 3.91, 95% CI 1.19-12.79, *P*=.02; ¥>100,000 group: OR 4.63, 95% CI 1.41-15.27, *P*=.01).

**Table 6:** Factors associated with telemedicine use by logistic regression analysis (N=514).

Variables			Univariate model					Multivariate model			
			OR^a^ (95% CI)		*P* value		OR (95% CI)			*P* value	
**Sex**											
	Male^b^	N/A^c^		N/A		N/A			N/A		
	Female	1.73 (1.12-2.67)		.01		1.56 (0.95-2.56)			.08		
**Age (y)**											
	≥60^b^	N/A		N/A		N/A			N/A		
	40-59	4.94 (2.19-11.17)		<.001		4.35 (1.84-10.29)			.001		
	18-39	11.45 (4.88-26.90)		<.001		9.20 (3.40-24.88)			<.001		
**Education**											
	Junior middle school or less^b^	N/A		N/A		N/A			N/A		
	High school	2.47 (1.15-5.29)		.02		2.45 (1.05-5.73)			.04		
	University or more	4.01 (1.98-8.14)		<.001		2.63 (1.11-6.23)			.03		
**Diabetes history (y)**											
	<1^b^	N/A		N/A		N/A			N/A		
	1-5	1.65 (0.86-3.15)		.13		1.21 (0.57-2.55)			.62		
	6-10	1.26 (0.72-2.22)		.42		1.25 (0.55-2.83)			.59		
	>10	1.08 (0.59-2.0)		.80		1.70 (0.76-3.84)			.20		
**Residence**											
	Urban^b^	N/A		N/A		N/A			N/A		
	Rural	0.95 (0.55-1.64)		.86		1.61 (0.82-3.19)			.17		
**Annual family income^d^**											
	¥<10,000^b^	N/A		N/A		N/A			N/A		
	¥10,000-¥50,000	3.44 (1.16-10.20)		.03		3.90 (1.21-12.51)			.02		
	¥50,000-¥100,000	4.18 (4.14-12.38)		.01		3.91 (1.19-12.79)			.02		
	¥>100,000	6.17 (2.10-18.11)		.001		4.63 (1.41-15.27)			.01		
**Diabetes type**											
	T1DM^b,e^	N/A		N/A		N/A			N/A		
	T2DM^f^	0.49 (0.28-0.85)		.011		0.98 (0.50-1.92)			.95		
	Others	1.72 (0.59-5.05)		.32		2.15 (0.62-7.48)			.23		
	Unknown	0.90 (0.39-2.08)		.81		1.51 (0.54-4.22)			.43		
**Vaccinated for COVID-19**											
	No^b^	N/A		N/A		N/A			N/A		
	Yes	1.58 (0.69-3.63)		.28		1.34 (0.54-4.22)			.53		
**COVID-19 infection**											
	No^b^	N/A		N/A		N/A			N/A		
	Yes	1.32 (0.85-2.04)		.22		0.92 (0.53-1.59)			.76		
**BI^g^ score**											
	Low BI^b^ (range 3-9)	N/A		N/A		N/A			N/A		
	High BI (range 10-15)	2.24 (1.37-3.66)		.001		1.98 (1.17-3.35)			.01		

^a^OR: odds ratio.

^b^Reference group.

^c^N/A: not applicable.

^d^CNY ¥1=US $0.1398.

^e^T1DM: type 1 diabetes mellitus.

^f^T2DM: type 2 diabetes mellitus.

^g^BI: behavioral intention.

## Discussion

### Determinants of BI to Use Telemedicine

Our study found that SI was the most important determinant of BI to use telemedicine in patients with diabetes, which is consistent with our previous study on the determinants of patients’ intentions to use diabetes management apps [33]. The study by Hennemann et al [41] also found that SI was the most important determinant of patients’ acceptance of web-based aftercare. The study by Alaiad and Zhou [32] replicated this finding in home health care robots. Diabetes is a chronic disease that requires long-term follow-up. The medical behavior intentions of patients with diabetes are inclined to be affected by the advice of their health care professionals, patients with the same disease, and family members’ support [42]. Tsai et al [43] found that the trust in family members was an important factor for older adult patients with diabetes to continue to choose telemedicine. Burden in the use of telemedicine and, in particular, the shortage of medical resources in mainland China has greatly restricted its recommendation of telemedicine to patients [44]. The introduction of artificial intelligence into telemedicine shows the potential to reduce the burden on health care professionals and improve telemedicine efficiency. Moreover, inadequate or no reimbursement remains an obstacle to the wider recommendation of telemedicine [45]. In addition, high-quality clinical research on the effectiveness of telemedicine in diabetes management is limited [46]. Evidence that medical staff recommend telemedicine to patients is insufficient [44,47]. Therefore, telemedicine should be included in the scope of hospital performance assessment and additional high-quality clinical research should be conducted to provide sufficient evidence for medical staff to recommend telemedicine to patients.

PE and FCs had a moderate impact on the BIs of patients with diabetes to use telemedicine. Our survey also found that the main concern of patients with diabetes using telemedicine was effectiveness, followed by safety. The research by Hoque and Sorwar [15] on the willingness of older adults to use mHealth found that PE was the most important determinant of BI. The study by Dou et al [26] on the BI of patients with hypertension to use mobile apps for hypertension management yielded similar findings. If patients with diabetes perceive that using telemedicine is effective for glycemic control, saves travel time, and can reduce the risk of infectious diseases such as COVID-19, they may be more willing to use telemedicine. However, the study by Scott et al [48] of telemedicine in patients with type 1 diabetes found that a remarkable decline occurred in the proportion of patients who were willing to continue with telemedicine beyond the pandemic. Therefore, we should select the most effective telemedicine model and platform so that patients with chronic diseases such as diabetes can perceive the benefits of telemedicine. There are many platforms and modes of telemedicine, such as telephone, videoconference, web portal, mobile app, wearable technology, and SMS text messaging [49]. Diabetes management mobile apps connected to Internet of Things devices show potential as an effective method for administering diabetes telemedicine, and many studies have confirmed the effectiveness of this model [6,50,51]. Patients can upload their health monitoring data at home (eg, blood sugar value) to the telemedicine platform through Internet of Things technology. Medical staff can remotely monitor patients’ health data, guide drug adjustment, and provide diabetes education and support. More patient-centered telemedicine models require further investigation.

The study by Wang et al [29] on consumer acceptance of health care wearable devices found that FCs positively influenced BI. The study by Lee et al [34] on patients’ emergency use intentions for mHealth services in Taiwan also found this relationship. Although smartphones have been popularized in China and the country encourages qualified hospitals to offer telemedicine services, medical resources and telemedicine services in low-income countries are relatively limited, and many patients with diabetes may not know how to find telemedicine platforms [3]. Telemedicine departments should be established to provide ongoing technology and internet support.

Our study did not find a positive impact of EE on BI, which was consistent with our previous web-based survey on the willingness of patients with diabetes to use diabetes management mobile apps [33]. The study by Dou et al [26] on hypertensive patients’ acceptance of mHealth technology for hypertension management and the research by Jewer [28] regarding patients’ intention to use web-based postings of emergency department wait times also did not find this impact. The possible reasons are related to the differences in the ages, education levels, and technology proficiencies of the investigated population and the complexity of the investigated technology. For example, patients, such as older adults who are unskilled in the use of telemedicine technologies may find EE to be an important determinant of BI [52,53]. Our research was based on the WeChat network, and the respondents may have a high proficiency in using social apps. This might be the reason we did not find a significant impact of EE on BI.

Our study did not find an influence of perceived COVID-19 susceptibility or perceived COVID-19 severity on BI, and we found no significant difference in the willingness and behaviors of telemedicine use between patients who had been vaccinated and those who had not been vaccinated or between those who had been infected with COVID-19 and those who had not been infected with COVID-19. A possible reason is that China has popularized its knowledge of COVID-19 through various channels. Before the change in COVID-19’s defense strategy, China publicized Omicron’s greater infectivity and lower severity in the official media. Therefore, the individual heterogeneity of the perceptions of COVID-19 susceptibility and PSE among the patients with diabetes was not significant, so it did not have a significant impact on BIs. Additional research is required to determine this relationship.

### Demographic Characteristics Associated With Telemedicine Use in Patients With Diabetes

We divided the sample into 2 groups (a low BI group and a high BI group). Univariate logistic regression analysis found that age, gender, education, family income, and BI were associated with telemedicine use. There were no significant correlations among the use of telemedicine and residence, diabetes duration, type of diabetes, whether the patients were COVID-19 vaccinated, or whether they had been infected with COVID-19. We then entered all the variables in the multivariate analysis to obtain the multivariable adjusted ORs and found that age, education, family income, and BI score were still related to patients’ telemedicine use. Previous studies on telemedicine also indicate that the use of telemedicine is higher for young patients and patients with higher education [3,21-23,54]. After adjusting for the BI of patients to use telemedicine, our study found that the use of telemedicine was higher in younger patients, those with higher education levels, and those with higher family income. A possible reason is that young patients and highly educated patients can access more telemedicine resources and there are fewer barriers to its actual use; thus, it may be easier for them to take action after they have an intention to use telemedicine. Horrell et al’s [21] survey of telemedicine use in patients with chronic conditions during COVID-19 found a higher proportion of individuals in households earning more than US $100,000 engaged in telehealth than those earning less than US $30,000. A survey in Korea also found that households with a monthly household income of ≥US $ 6000 had higher odds of approving telemedicine [22]. Because telemedicine is not included in insurance reimbursement programs in most regions, patients with low family income may not use telemedicine because of economic constraints, even if they show BIs. Thus, telemedicine should be included in insurance programs in the future. The correlation between sex and telemedicine use has been inconsistent across studies [21,23]. In our study, univariate logistic regression analysis showed that telemedicine use was higher among female patients than among male patients. However, after adjusting for multiple variables, such as BI, we found no correlation between sex and telemedicine use.

### Strengths and Limitations

Our study was the first to investigate the telemedicine use behavior of patients with diabetes after China lifted most of its COVID-19 restrictions. We confirmed the UTAUT model using telemedicine in patients with diabetes in China. We identified the determinants of BI to use telemedicine and analyzed the demographic characteristics associated with telemedicine use in patients with diabetes, which are important for the promotion of telemedicine in the post–COVID-19 pandemic era.

However, our study had several limitations. First, our survey was based on the WeChat network, which might induce potential selection bias. However, the sample representation is not essential in causal inference analysis [55,56], and our study could inform research on factors influencing the BIs and use behaviors of telemedicine in patients with diabetes. Second, the sample size of subgroups for some of the characteristics in our study was insufficient. Larger samples are required for further investigation. Finally, our survey was a cross-sectional survey. Patients currently have BIs, but their conditions may not be suitable for internet treatment at present. In the future, prospective studies are needed to observe the correlation between BIs and other relevant factors and the use of telemedicine.

### Conclusions

SI, PE, and FCs positively affected the BIs of patients with diabetes to use telemedicine. After adjusting for BI, young patients, highly educated patients, and patients with a high family income used telemedicine more frequently. We need to take action to promote BI and pay special attention to the needs of older adult patients, patients with low income, and patients with low levels of education.

## Abbreviations

AVE: average variance extracted
BI: behavioral intention
EE: effort expectancy
FC: facilitating condition
mHealth: mobile health
OR: odds ratio
PE: performance expectancy
PSE: perceived severity
SI: social influence
UTAUT: Unified Theory of Acceptance and Use of Technology
